# TMP19: A Novel Ternary Motif Pattern-Based ADHD Detection Model Using EEG Signals

**DOI:** 10.3390/diagnostics12102544

**Published:** 2022-10-20

**Authors:** Prabal Datta Barua, Sengul Dogan, Mehmet Baygin, Turker Tuncer, Elizabeth Emma Palmer, Edward J. Ciaccio, U. Rajendra Acharya

**Affiliations:** 1 School of Management & Enterprise, University of Southern Queensland, Toowoomba, QLD 4350, Australia; 2 Faculty of Engineering and Information Technology, University of Technology Sydney, Sydney, NSW 2007, Australia; 3 Department of Digital Forensics Engineering, Technology Faculty, Firat University, 23119 Elazig, Turkey; 4 Department of Computer Engineering, Faculty of Engineering, Ardahan University, 75000 Ardahan, Turkey; 5 Centre of Clinical Genetics, Sydney Children’s Hospitals Network, Sydney 2031, NSW, Australia; 6 School of Women’s and Children’s Health, University of New South Wales, Sydney NSW 2031, Australia; 7 Department of Medicine, Columbia University Irving Medical Center, New York, NY 10032, USA; 8 Department of Electronics and Computer Engineering, Ngee Ann Polytechnic, Singapore 599489, Singapore; 9 Department of Biomedical Engineering, School of Science and Technology, SUSS University, Singapore 599494, Singapore; 10 Department of Biomedical Informatics and Medical Engineering, Asia University, Taichung 41354, Taiwan

**Keywords:** ternary motif pattern, ADHD detection, EEG signal classification, signal processing

## Abstract

Attention deficit hyperactivity disorder (ADHD) is a common neurodevelopmental condition worldwide. In this research, we used an ADHD electroencephalography (EEG) dataset containing more than 4000 EEG signals. Moreover, these EEGs are noisy signals. A new hand-modeled EEG classification model has been proposed to separate healthy versus ADHD individuals using the EEG signals. In this model, a new ternary motif pattern (TMP) has been incorporated. We have mimicked deep learning networks to create this hand-modeled classification method. The Tunable Q Wavelet Transform (TQWT) has been utilized to generate wavelet subbands. We applied the proposed TMP and statistics to construct informative features from both raw EEG signals and wavelet bands by generating TQWT. Herein, features have been generated by 18 subbands and the original EEG signal. Thus, this model is named TMP19. The most informative features have been chosen by deploying neighborhood component analysis (NCA), and the selected features have been classified using the k-nearest neighbor (kNN) classifier. The used ADHD EEG dataset has 14 channels. Thus, these three phases—(i) feature extraction with TQWT, TMP, and statistics; (ii) feature selection by deploying NCA; and (iii) classification with kNN—have been applied to each channel. Iterative hard majority voting (IHMV) has been applied to obtain a higher and more general classification response. Our model attained 95.57% and 77.93% classification accuracies by deploying 10-fold and leave one subject out (LOSO) cross-validations, respectively.

## 1. Introduction

Attention deficit hyperactivity disorder (ADHD) is a persistent neurodevelopmental disorder [1,2,3]. It can increase the risk of poor educational and occupational outcomes, social disability, and other psychiatric conditions [4]. ADHD is often diagnosed between the ages of 3 and 7 years, but may not be recognized until adulthood [5,6]. ADHD symptoms include inattentiveness and hyperactivity-impulsivity [7]. People with ADHD often have additional features such as impairments in their executive function and emotional regulation [8,9,10]. Systematic reviews show that ADHD affects 5% of children and adolescents and 2.5% of adults globally [11]. Boys are more likely to be diagnosed with ADHD than girls [12,13]. Early diagnosis is very important for this condition to optimize management and reduce the long-term negative sequalae in psychosocial wellbeing and integration into the community [14]. Management usually combines patient and family education and pharmacological and non-pharmacological interventions such as a range of behavioral and neurocognitive therapies [15]. The aim is to eliminate disease symptoms. Psychologists diagnose ADHD, and the professionals have responsibility for assessment and diagnosis. This assessment typically involves a combination of standardized evaluation scales plus a clinical interview, compared to the DSM-5 or ICD (International Statistical Classification of Diseases and Related Health Problems) diagnostic criteria [16]. Diagnosing ADHD can be challenging, particularly in regions with limited access to suitably trained health professionals.

There is, therefore, interest in the ability of other diagnostic tools to improve equitable access to an early diagnosis [17]. One suggested diagnostic data type is the EEG [18]. The FDA has stated that the EEG may be a helpful diagnostic tool in ADHD diagnoses [19]. An increasing number of studies on the potential diagnostic utility of EEG have now been reported in the literature. This paper proposes a new hand-modeled EEG classification for the automatic interpretation of EEG signals for diagnosing ADHD in children. The model developed in this study has high classification success and low computational complexity.

The diagnosis of ADHD using EEG signals remains a hot topic with as yet no universally agreed upon guideline for the diagnostic use and interpretation of the EEG. Moghaddari et al. [20] used the Convolutional Neural Network (CNN) for ADHD diagnosis in children. Their study utilized EEG signals from 31 ADHD and 30 healthy children, which were preprocessed and free of noise and artifacts. Thereafter, the EEG signals were converted to RGB images and provided as input to a 13-layer CNN. The developed model was evaluated with the 5-fold cross-validation technique, and an average accuracy of 99.06% was achieved. An ADHD classification method using power spectral density (PSD), spectral entropy (SE), and long short-term memory (LSTM) methods has been proposed by Tosun [21]. Their study applied an 80:20 hold-out validation strategy, and the proposed method yielded a 92.15% accuracy. Khoshnoud et al. [22] performed a nonlinear EEG analysis in children with ADHD. The largest Lyapunov exponent (LLE) and approximate entropy (ApEn) methods were used to obtain the nonlinear characteristics of the signal. These features were evaluated with a Probabilistic Neural Network (PNN), and an 87.5% classification accuracy was obtained. Chen et al. [23] proposed a deep learning framework to identify children with ADHD. In their proposed method, EEG signals are converted into image data and given as input to the CNN developed in the study. EEG signals from 50 children with ADHD and 51 healthy children were utilized. The developed model provided a 94.67% accuracy performance on the test data. Tenev et al. [24] proposed a learning approach for classifying adult ADHD individuals automatically. Their study analyzed EEG signals from 117 adults (67 ADHD and 50 controls) using a Support Vector Machine (SVM) and voting method. The proposed method achieved an 82.3% success rate in separating ADHD versus control groups. Saini et al. [25] developed an EEG signal-based machine learning model for ADHD diagnosis. The developed model uses principal component analysis (PCA) for feature selection and k-nearest neighbor (kNN) for classification. In this study, EEG signals from 77 ADHD and 80 healthy children were classified, and an accuracy of 86% was achieved. Dubreuil-Vall et al. [26] collected EEG signals from 40 subjects (20 ADHD and 20 controls) for ADHD classification. In their study, firstly, the signal was preprocessed and then the spectrogram of the signal was extracted. Thereafter, the spectrogram images of the EEG signal were fed as input to the custom designed CNN, and classification was made. The developed model reached an 88% classification accuracy. Tor et al. [27] used empirical mode decomposition (EMD) and discrete wavelet transform (DWT) decomposition methods and nonlinear features for ADHD, conduct disorder (CD), and ADHD+CD detection. They analyzed EEG data of 123 children (45 ADHD, 62 conduct disorder + ADHD, and 16 conduct disorder subjects) with a kNN classifier and obtained an accuracy of 97.88%. Loh et al. [28] reviewed the automated ADHD detection methods published in the last decade. They reviewed all the machine learning and deep learning techniques employed for the automated detection of ADHD using both physiological signals and images.

The essential motivations of the presented model are:

- Presenting a new motif pattern to generate textural features;
- Proposing a hand-modeled one-dimensional signal classification architecture;
- Attaining robust and high classification performance with low time complexity.

Thus, this study has three main motivations. First, motifs of signals are very useful for extracting features. A new feature generation model has been proposed to use motifs as a feature vector, and this feature extraction function is named TMP. We aimed to present a highly accurate feature engineering model. Hence, we used both hand-crafted features and an architecture miming a deep learning architecture to attain high classification accuracy. Deep learning models have multiple levels/layers to generate distinctive features. Thus, we have used a multileveled feature extraction model. By using this model, an accurate EEG signal classification method has been proposed. To demonstrate its robustness, we used two validation techniques (10-fold CV and LOSO CV). These validations yielded 95.57% and 77.93% classification accuracies by deploying 10-fold CV and LOSO CV, respectively.

The novelties and contributions are:

- A new ternary motif pattern has been proposed in this research. The main objective of the proposed TMP is to extract hidden and informative features from EEG signals.
- A new generation multilevel feature engineering model has been proposed in this research to attain high classification accuracy on the EEG dataset. The presented feature engineering model uses 19 levels and TMP to extract features. Thus, this model is named TMP19.
- In the feature selection phase, the main feature extraction function is NCA. At the same time, we employed threshold-based elimination to obtain more distinctive features. Thus, the model is named threshold–based NCA.
- We integrated a noisy EEG dataset containing 4173 EEG signals, each of four seconds in length. We believe this is the first proposal of a classification model for this dataset in the literature.
- Our proposal—TMP19—was tested using two validation techniques and showed robust results. The TMP19 attained 95.57% and 77.93% classification accuracies by deploying 10-fold CV and LOSO CV, respectively.

## 2. Material and Method

### 2.1. Material

We utilized a noisy EEG dataset in this work [29,30]. The dataset contains 2330 healthy EEG signals and 1843 ADHD EEG signals. The lengths of these EEG signals are equal and four seconds in length. In this EEG dataset, there are 14 channels. The frequency sampling of these EEG signals is 128 Hz.

### 2.2. Method

**Figure 1:**
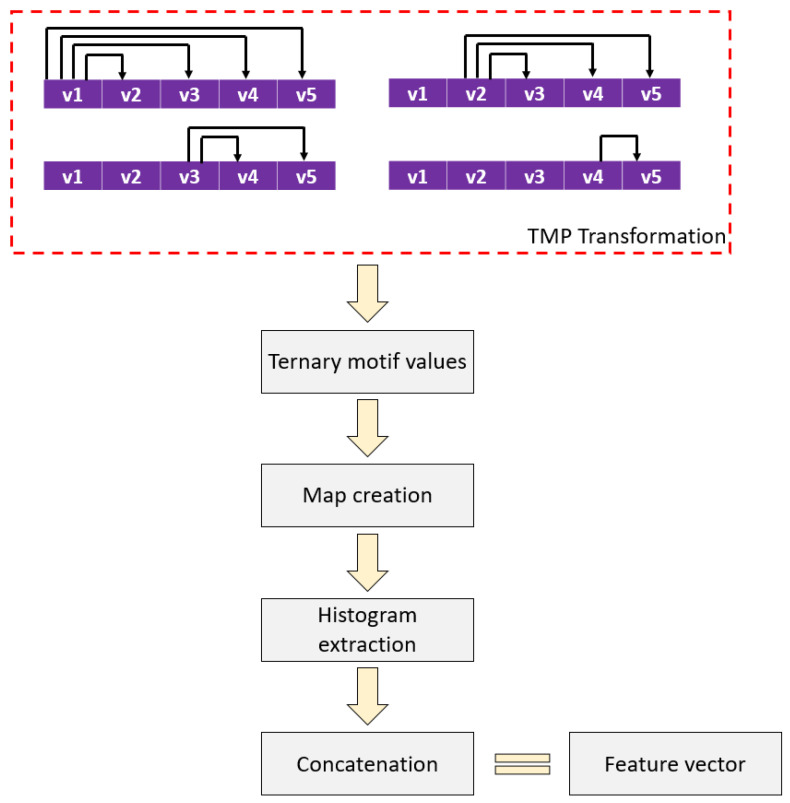
Model overview of the proposed TMP feature extraction function.

In this paper, a new local feature extraction function has been proposed, named the ternary motif pattern. Herein, we integrated five-sized overlapping blocks to obtain motifs, and we applied the ternary function to find upper, equal, and lower values. Two feature maps have been generated using these values, and histograms of these feature maps have been utilized as feature vectors. The model overview of the presented TMP is demonstrated in Figure 1.

To better explain this model, detailed descriptions have been given below.

*Step 1:*  Create overlapping blocks with a length of five.

(1) $$bl^{t}=S\left(t:t+4\right),\;t\in \left\{1,2,\ldots ,len-4\right\}$$

Herein, $bl^{t}$ is the overlapping block with a length of five, and len defines the length of the signal.

*Step 2:*  Generate ternary motifs.

(2) $$val_{k}=tf\left(bl_{i}^{t},bl_{j}^{t}\right),\;i\in \left\{1,2,3,4\right\},\;j\in \left\{i+1,i+2,\ldots ,5\right\},\;k\in \left\{1,2,\ldots ,10\right\}$$

(3) $$tf\left(bl_{i}^{t},bl_{j}^{t}\right)=\left\{\begin{array}{c} 0,bl_{i}^{t}<bl_{j}^{t}\\ 1,bl_{i}^{t}=bl_{j}^{t}\;\\ 2,\;bl_{i}^{t}>bl_{j}^{t}\; \end{array}\right.$$

where val represents ternary motif values, and tf() defines ternary function.

*Step 3:*  Calculate map values using ternary motif values.

(4) $$m_{t}^{1}=\overset{5}{\underset{j=1}{\sum }}val_{j}\times 3^{j-1}$$

(5) $$m_{t}^{2}=\overset{5}{\underset{j=1}{\sum }}val_{j+5}\times 3^{j-1}$$

The symbols $m^{1}$ and $m^{2}$ represent the first and second map signals, respectively.

*Step 4:*  Extract histograms of map signals.

(6) $$h^{1}=\vartheta \left(m^{1}\right)$$

(7) $$h^{2}=\vartheta \left(m^{2}\right)$$

where $h^{1}$ and $h^{2}$ are histograms of first and second maps. The length of each map signal is 243 (=3^5^).

*Step 5:*  Merge the generated histograms and obtain a feature vector.

(8) $$fv\left(q\right)=h^{c}\left(q+243\times \left(c-1\right)\right),\;c\in \left\{1,2\right\},\;q\in \left\{1,2,\ldots ,243\right\}$$

The symbol fv defines the generated feature vector with a length of 486 (=243 × 2).

These five steps above define the proposed TMP.

We suggested a new hand-modeled signal classification method in this paper, and our proposal is named TMP19. TMP19 contains four main phases, and these phases are:

- Feature extraction;
- Feature selection;
- Classification;
- Majority voting.

**Figure 2:**
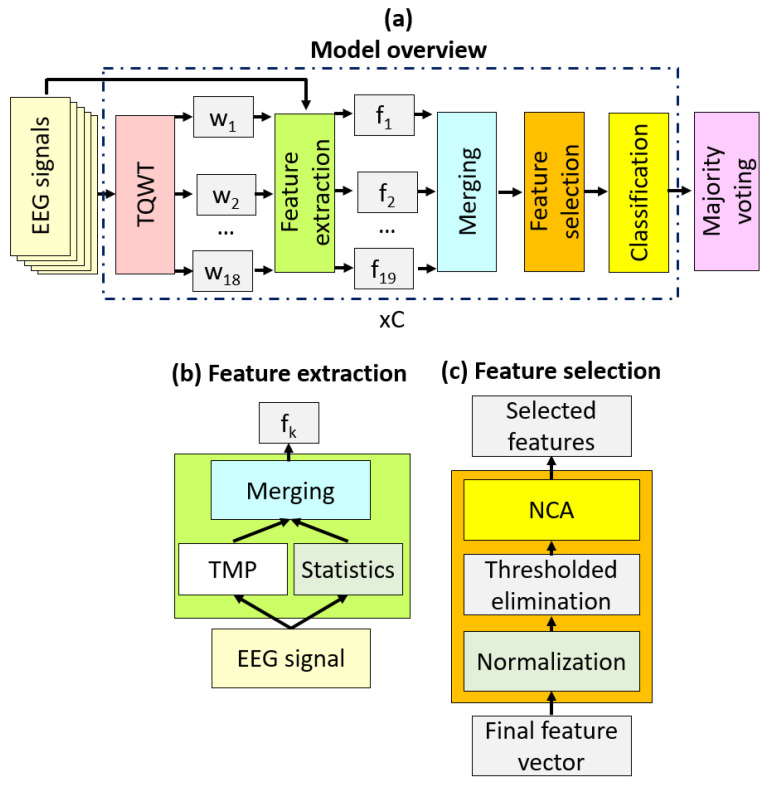
(**a**) Overview of the TMP19 signal classification architecture, (**b**) the proposed statistical and TMP-based feature extraction method, and (**c**) feature selection with the thresholded NCA method.

In Figure 2, a graphical overview of the proposed TMP19 is demonstrated.

**Figure 3:**
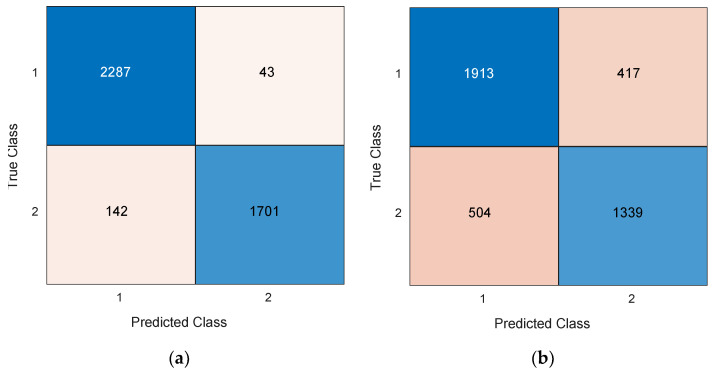
Generated confusion matrices by TMP19 on the ADHD EEG dataset by deploying (**a**) 10-fold CV, (**b**) LOSO CV. Herein, the used classes are enumerated as 1 and 2. These numbers are defined as healthy and ADHD classes. 1: Healthy, 2: ADHD.

Figure 2 depicts our proposed TMP19 architecture. In Figure 3, C represents the number of channels. Phases of this model are defined below, briefly describing the TMP19 EEG signal classification model.

#### 2.2.1. Feature Extraction

In the feature extraction phase, we utilized three methods: (i) TQWT [31], (ii) the proposed TMP feature extraction function, and (iii) statistical feature generator. First, we generated wavelet bands using TQWT transformation. TQWT is a parametric and third-generation wavelet transform. By using redundancy (r), oscillation (Q), and the number of level (J) parameters, a variable multileveled wavelet transform was created. The high oscillatory wavelet transform was assigned 4 and 3 for the Q and r parameters.

The system’s main aim was to propose a new feature engineering model as in DarkNet19. Thus, we generated 18 wavelet coefficient bands to use 19 inputs. These 19 signals (raw EEG signal + 18 wavelet bands) have been utilized as inputs to the feature extraction functions. The system contains two feature extraction functions: the TMP and the statistical feature generator. Both statistical parameters and motifs have been extracted using these feature extractors in the space and frequency domains. The steps of the proposed feature extraction method are given below.

**Step 1:**  Apply TQWT to the EEG signal for generating wavelet bands.

(9) $$R=\tau \left(S,4,3,17\right)$$

where R defines TQWT bands, S is the EEG signal, 4, 3, and 18 are the Q, r, and J parameters of the TQWT function (τ()).

**Step 2:**  Generate features using TMP and statistical feature generation function.

(10) $$f_{1}=concat\left(TMP\left(S\right),SG\left(S\right)\right)$$

(11) $$f_{l+1}=concat\left(TMP\left(R_{l}\right),SG\left(R_{l}\right)\right),\;l\in \left\{1,2,\ldots ,18\right\}\;$$

In this step (Step 2), 19 feature vectors have been generated by deploying the TMP feature generator (TMP()), and statistical generator (SG()). TMP() function generates 486 features from a one-dimensional signal, and SG() extracts 14 features from a signal since 14 statistical moments [32] have been used in this function. These moments are (i) maximum, (ii) minimum, (iii) average, (iv) variance, (v) standard deviation, (vi) median, (vii) range, (viii) root mean square, (ix) energy, (x) Shannon entropy, (xi) sure entropy, (xii) log energy entropy, (xiii) threshold entropy, and (xiv) mean absolute deviation. concat() is concatenation function and it is feature merging function. By using Step 2, 19 feature vectors have been created and the length of each feature vector is 500 (=486 + 14).

**Step 3:**  Concatenate the 19 generated feature vectors to create the final feature vectors.

(12) $$f_{g}\left(p\right)=X\left(p+500\times \left(g-1\right)\right),\;p\in \left\{1,2,\ldots ,500\right\},\;g\in \left\{1,2,\ldots ,19\right\}$$

Herein, X defines the merged feature vector with a length of 9500 (=500 × 19).

#### 2.2.2. Feature Selection

In this research, we proposed an improved NCA-based protocol [33], with the feature selection method being named the threshold-based NCA. The selector has two layers: (i) threshold-based redundant feature elimination and (ii) NCA-based feature selection. In this phase, we selected the most informative and valuable 250 features from the generated 9500 features. The steps of this phase are:

**Step 4:**  Normalize the final feature vector by deploying a min-max normalization.

(13) $$X^{N}=\frac{X-\min\left(X\right)}{\max\left(X\right)-\min\left(X\right)}$$

where $X^{N}$ defines the normalized final features, and min() and max() are the minima and maximum value finding functions, respectively.

**Step 5:**  Eliminate the redundant features using a threshold value.

(14) $$ag_{d}=\overset{n}{\underset{i=1}{\sum }}X^{N}\left(d,i\right),d\in \left\{1,2,\ldots ,dim\right\}\;$$

(15) $$nX\left(:,ct\right)=X\left(:,i\right),\;if\;ag_{d}>th$$

Herein, the summarization value of each vector ($ag_{d}$) has been calculated in Equation (14), where dim is the number of EEG signals. In Equation (15), features are eliminated, and a new final feature vector (nX) has been generated using a threshold value (th). In this research, th is selected as zero.

**Step 6:**  Calculate qualified/sorted indexes by deploying the NCA feature selection function.

(16) $$id=\xi \left(nX,y\right)\;$$

where id is a sorted index vector with a length of 9500, y defines real outputs/labels, and ξ() is the NCA feature selection function.

**Step 7:**  Select the most meaningful 250 features from the nX.

(17) $$sX\left(d,i\right)=nX\left(d,id\left(i\right)\right),\;i\in \left\{1,2,\ldots ,250\right\}$$

Herein, sX is a selected feature vector with a length of 250.

#### 2.2.3. Classification

To obtain classification accuracy and the predicted vector of each channel, we used a simple/shallow classifier. The kNN [34] classifier has been used, which is named the Weighted kNN. The hyperparameters of the Weighted kNN are:

k:10;

Distance weight: squared inverse;

Distance: L1-norm (Manhattan);

Validation: 10-fold CV/LOSO CV.

As is evident from the above description, we used two validation techniques (10-fold CV and LOSO CV) to calculate the robust results obtained with this classifier. The classification step is given below.

**Step 8:**  Calculate the predicted vector of each channel by applying the kNN classifier.

(18) $$p_{c}=\kappa \left(sX,y\right),\;c\in \left\{1,2,\ldots ,14\right\}\;$$

where $p_{c}$ represented predicted vectors of the cth channel, and κ() is the defined kNN classifier.

**Step 9:**  Repeat Steps 1–8 by the number of channels. We used an ADHD dataset containing 14 channels. Thus, we repeated these steps 14 times.

#### 2.2.4. Majority Voting

In the majority voting phase, the iterative hard majority voting (IHMV) algorithm was used to obtain the best classification result. IHMV was proposed by Dogan et al. [35] in 2021. We calculated 14 predicted vectors from the EEG channels individually. By incorporating the 14 predicted vectors, 12 (=14 − 2) new voted predicted vectors were created for each validation technique. The steps of this phase are listed below.

**Step 10:**  Calculate the classification accuracy of each channel.
**Step 11:**  Qualify/sort the predicted vectors using the predicted vectors and obtain qualified/sorted indexes.

(19) $$ix=sort\left(p\right)\;$$

where ix defines indexes of the sorted predicted vectors.

**Step 12:**  Calculate voted predicted vectors by deploying the mode function.

(20) $$v_{r-2}=\omega \left(p_{ix_{1}},p_{ix_{2}},\ldots ,p_{ix_{r}}\right),r\in \left\{3,4,\ldots ,C\right\}\;\;$$

Herein, v represents the voted vector, C represents the number of channels, and ω() defines the mode function. In these steps, 12 voted vectors have been generated.

**Step 13:**  Calculate classification accuracies of the voted vectors.
**Step 14:**  Choose the most accurate voted vector as the final predicted vector.

The given 14 steps above define the proposed TMP19 feature engineering model.

## 3. Results

We have proposed a new feature engineering model that uses four phases in this research. These phases use lightweight methods. Thus, the proposed TMP19 is a lightweight signal classification model, and there is no need to use expensive hardware. We implemented this model on a simply configured computer (main memory: 16 GB, processor: Intel i7-7700, operating system: Windows 11, programming environment: MATLAB 2021a). We used two validation techniques. Moreover, channel-wise voted results have been given in this section.

### 3.1. Performance Metrics

The used ADHD EEG dataset contains two classes, ADHD and normal. Thus, this is a binary classification problem. We built in two common classification performance measures: classification accuracy and geometric mean. To assess these metrics, the number of true positives (tp), false positives (fp), true negatives (tn), and false negatives (fn) are deployed. The mathematical notations of the accuracy (acc) and geometric mean (gm) are given below.

(21) $$acc=\frac{tp+tn}{tp+fp+tn+fn}$$

(22) $$gm=\sqrt{\frac{tp}{tp+fn}\times \frac{tn}{tn+fp}}$$

### 3.2. Channel-Wise Results

The used EEG dataset contains 14 channels. To comprehensively depict the proposed model’s classification performance, we applied our proposal to each channel, and the classification performances of each channel were calculated. Furthermore, two validations were used in the classification phase. The channel-wise results are tabulated in Table 1.

Table 1 highlights the best results using a bold font face. By applying a 10-fold CV, the most accurate channel is the 13th since our TMP19 model yielded 92.16% classification accuracy and 91.91% geometric mean. According to LOSO CV results, the best classification accuracy is 74.84% on the 7th channel, and the best geometric mean attained on the 13th channel by employing LOSO CV.

### 3.3. Voted Results

In the last phase of the TMP19, the majority voting component is availed of. We applied the IHMV algorithm in this phase to create C-2-voted vectors. Twelve voted vectors were generated by deploying the IHMV algorithm. The results of the voted vectors are listed in Table 2.

Table 2 shows that the best classification accuracies were obtained by using 11th and 1st voted vectors when deploying 10-fold CV and LOSO CV, respectively. Per Table 2, IHMV increased the classification accuracies from 92.16% and 74.84% to 95.57% and 77.93% by deploying 10-fold CV and LOSO CV, respectively. These results are the final results of the proposed TMP19.

### 3.4. Final Results

Our proposed TMP19-based model generates 26 (=14 results from 14 channels + 12 voted results) results, and this model selects the best results among the 26 generated results. Thus, the suggested TMP19 is a self-organized feature engineering model. Moreover, two validations were applied in the classification phase. The calculated confusion matrices of the presented TMP19 by deploying LOSO CV and 10-fold CV are given in Figure 3.

As can be seen from Figure 3, our proposed TMP19 attained 95.57% and 77.93% classification accuracies on the ADHD dataset with a 10-fold CV and LOSO CV, respectively.

## 4. Discussion

A new feature engineering model has been proposed in this research by mimicking a deep learning structure. The main motivation of the proposed TMP19 is to extract hidden motifs to obtain high classification performances. The TMP19 generates features at both high levels and low levels. High-level features have been generated using wavelet bands. Moreover, features at the frequency domain have been extracted. The threshold-based NCA method has been used to obtain distinctive features. kNN has been employed to show the classification capability of the generated features. The best classification results were generated and selected from the model by applying a majority voting technique. Then the channel-wise and voted results were given. Per the results, the most appropriate channels are the 7th and 13th for ADHD detection. Our model attained a 95.57% classification accuracy by deploying a 10-fold CV and a 77.93% classification accuracy using LOSO CV. These results showed that the presented TMP19 is a successful EEG classification model. Furthermore, we believe that we are the first team to use this dataset to develop a machine-learning model.

The results of other state-of-art classification models and our TMP19 were compared to highlight the classification ability of our model. These results are listed in Table 3.

As can be seen from Table 3, our model produced better accuracy than the results of other state-of-the-art methods. Tor et al. [27] achieved better accuracy than our study. They used 3 classes with 123 subjects in the study. Classes include 45 ADHD, 62 conduct disorder + ADHD, and 16 conduct disorder subjects, and the amount of data for each class is small. In addition, two different validation techniques were used in this study. These are the 10-fold CV and the LOSO CV, respectively. Another advantage is that noisy EEG signals were used in our study. A superior side of our study to other previously presented works is to use LOSO CV. Since reliable results have been calculated using LOSO CV, we depicted our model’s performances in real-world applications by helping LOSO CV. Although our proposed method exhibits a low computational complexity, it outperforms. The results obtained show the superiority of the proposed method.

The benefits of the presented TMP:

- A novel feature generation function was introduced. This function generates motifs. Thus, this feature generator is named TMP.
- An accurate one-dimensional signal classification architecture has been proposed by using TMP. This model contains 19 levels. Thus, it is named TMP19.
- Simple methods have been used to create the TMP19 model. Thus, the implementation of this model is straightforward and of low complexity.
- TMP19 is a parametric model. Therefore, next-generation TMP-based classification models can be proposed by using different classification methods.
- TMP19 is a highly accurate model.
- The robustness of the presented TMP19 is demonstrated by deploying a 10-fold CV and LOSO CV.

Drawbacks:

- Parameters should be optimized to gain higher classification performances.
- Recently, authors in [37] have developed an automated system to detect ADHD and conduct disorder in children using empirical wavelet transform and entropy features extracted from electrocardiogram (ECG) signals. They obtained an accuracy of 88% in classifying ADHD, ADHD + CD, and CD patients for appropriate intervention using accessible ECG signals. In the future, ECG and heart rate variability (HRV) signals can be used for automated ADHD detection as they can be easily acquired using wearable devices.
- More disorders can be used to evaluate the performance of the TMP19 model.

## 5. Conclusions

ADHD detection using EEG signals has great potential in improving the equitable early diagnosis of ADHD, maximizing appropriate treatments, and minimizing the potential long-term negative impacts of this globally common neurodevelopmental condition. However, there is a lack of consensus on the best way to utilize EEG and how machine learning may be beneficial in optimizing this as a diagnostic tool. To overcome this problem, we proposed a new feature generator called TMP. The main goal of the TMP is to extract distinctive features from EEG signals. A feature engineering model was proposed by applying the proposed feature generator—TMP. This model has 19 levels in the feature extraction phase. Hence, it is named TMP19. TMP19 was applied to a noisy EEG signal dataset for the detection of ADHD. Moreover, we used two validation techniques to show the robustness of the proposed TMP19. As can be seen from Section 5, our TMP19 reached 95.57% and 77.93% classification accuracies using 10-fold CV and LOSO CV techniques, respectively.

We plan to develop an intelligent EEG signal classification application in future work. This application will extract information from EEG signals to detect ADHD and potentially other neurodevelopmental and mental health conditions. Moreover, we plan to devise and develop an intelligent brain cap that will detect changes automatically.

## Figures and Tables

**Table 1:** Channel-wise results (%) by deploying 10-fold CV and LOSO CV.

Channel	10-fold CV		LOSO CV	
	Accuracy	Geometric Mean	Accuracy	Geometric Mean
**1**	87.75	87.21	72.25	71.16
**2**	86.65	85.69	71.41	70.19
**3**	89.70	89.17	68.56	67.88
**4**	87.08	86.38	67.77	67.29
**5**	91.47	91.13	74.31	73.73
**6**	88.43	87.74	65.49	64.83
**7**	88.35	87.22	**74.84**	73.55
**8**	86.17	85.29	61.80	59.37
**9**	87.90	87.52	66.59	66.12
**10**	86.10	85.65	58.59	58.10
**11**	86.56	85.78	71	70.09
**12**	84.14	83.27	72.68	71.79
**13**	**92.16**	**91.91**	74.57	**74.35**
**14**	83.30	81.85	66.81	65.10
**General** **(mean ± SD)**	87.55 ± 2.45	86.36 ± 2.70	69.05 ± 4.89	68.11 ± 5.05

**Table 2:** Voted results (%) by deploying 10-fold CV and LOSO CV.

Voted Vector	10-fold CV		LOSO CV	
	Accuracy	Geometric Mean	Accuracy	Geometric Mean
**1**	94.49	94.25	**77.93**	**77.23**
**2**	93.24	92.45	75.39	73.46
**3**	94.49	94.11	77.45	76.89
**4**	93.96	93.30	75.17	73.28
**5**	95.21	94.89	77.38	76.74
**6**	94.20	93.57	75.41	73.81
**7**	95.16	94.77	76.95	76.35
**8**	94.49	93.88	75.58	74.13
**9**	95.40	95.03	76.56	75.86
**10**	94.94	94.40	74.89	73.62
**11**	**95.57**	**95.18**	75.99	75.23
**12**	94.90	94.32	74.84	73.41
**General** **(mean ± SD)**	94.67 ± 0.66	94.17 ± 0.79	76.12 ± 1.08	75 ± 1.54

**Table 3:** Summary of comparison of our results with other methods developed using EEG signals.

Author	Year	Method	Key Point(s)	Result(s) (%)
**Mohammadi et al.** [36]	2016	Preprocessing, nonlinear feature extraction (fractal dimension, LLE, ApEn), mRMR, and neural networks	- 60 subjects (30 ADHD, 30 control) - 70:10:20 hold-out validation	Acc. = 93.65
**Tenev et al.** [24]	2014	SVM and voting	-117 subjects (67 ADHD, 50 control) -10-fold CV	Acc. = 82.3
**Tosun** [21]	2021	Data augmentation, PSD, SE, and LSTM	- 16 subject - 80:20 hold-out validation	Acc. = 92.15
**Khoshnoud et al.** [22]	2015	Preprocessing, LLE, ApEn, PNN	- 22 subject (12 ADHD, 10 control) - 75:25 hold-out validation	Acc. = 87.5
**Chen et al.** [23]	2019	EEG signal to image conversion, CNN	- 101 subject (50 ADHD, 51 control) - 10-fold CV	Acc. = 94.67
**Saini et al.** [25]	2022	PCA and kNN	- 157 subject (77 ADHD, 80 control)	Acc. = 86.0
**Tor et al.** [27]	2021	Empirical mode decomposition, Discrete wavelet transform, kNN	- 123 subjects (45 ADHD, 62 conduct disorder + ADHD, 16 conduct disorder) - 10-fold CV	Acc.= 97.88
**Dubreuil-Vall et al.** [26]	2020	Preprocessing, spectrogram conversion and CNN	- 40 subject (20 ADHD, 20 control) - Leave pair out CV	Acc. = 88.0
**Our method**		TQWT, TMP19, NCA, kNN, and majority voting	- 121 subjects (61 ADHD, 60 control) - 10-fold CV and LOSO CV	10-fold CV
				Acc. = 95.57 Gm. = 95.18
				LOSO CV
				Acc. = 77.93 Gm. = 77.23

## Data Availability

The EEG dataset database has been downloaded from [29,30].
